# Influence of Carbonated Recycled Concrete Powder on Hydration, Shrinkage and Mechanical Performance of OPC-FA Systems

**DOI:** 10.3390/ma19102155

**Published:** 2026-05-21

**Authors:** Yuanchao Ou, Yanran Ma, Xinning He, Jing Ni, Yuanhao Fu, Congcong Wu, Dejian Wang, Yonghui Wang

**Affiliations:** 1 College of Civil and Hydraulic Engineering, Bengbu University, Bengbu 233000, China; ycou623@163.com (Y.O.);; 2 The Key Laboratory of Universities in Anhui Province for Prevention of Mine Geological Disasters, Anhui University of Science and Technology, Huainan 232001, China

**Keywords:** carbonated recycled concrete powder, hydration behavior, shrinkage, pore structure, nanoindentation

## Abstract

**Highlights:**

- Carbonated RCP (CRCP) modifies hydration and promotes early reaction at low dosages.
- CRCP significantly reduces autogenous and drying shrinkage of mortars.
- Pore structure is refined with fewer harmful pores with the incorporation of 8% CRCP.
- Improved strength is linked to denser C–S–H and higher elastic modulus.

**Abstract:**

The effective utilization of recycled concrete powder remains a key challenge for sustainable construction. In this study, carbonated recycled concrete powder (CRCP) was applied to replace cement at levels of 4–16% in Portland cement–fly ash (OPC-FA) systems, and its effects on fresh properties, hydration behavior, shrinkage, pore structure, and mechanical performance were systematically investigated. The incorporation of CRCP reduced flowability and accelerated setting, while slightly advancing and enhancing the main hydration peak at 4–8% replacement, accompanied by higher CH at early ages and increased C–S–H formation at later stages. More significantly, the addition of CRCP substantially decreased both autogenous and drying shrinkage, achieving reductions in the ranges of 6.0–21.4% and 3.2–24.1%, respectively. This improvement is primarily attributed to the elevated internal relative humidity and the lowered capillary pressure within the system. In addition, the mechanical properties exhibited a clear optimum with the addition of 8% CRCP, where the 28 d compressive strength and flexural strengths increased by 16.3% and 4.0%, respectively. Further analysis indicates that this improvement is associated with a higher fraction of high-modulus regions and an increase in average elastic modulus from 23.89 GPa to 27.42 GPa, reflecting a denser microstructure. These results demonstrate that CRCP can effectively regulate hydration and microstructure, providing a feasible approach for improving dimensional stability and mechanical performance while enabling the value-added utilization of recycled concrete powder.

## 1. Introduction

The global construction industry faces dual existential challenges: the continued production of construction and demolition waste (CDW) and the massive carbon footprint of ordinary Portland cement (OPC) manufacturing [1,2,3]. At present, more than 10 billion tons of CDW are generated worldwide each year, of which waste concrete accounts for over 50% [2,4]. However, this large amount of waste concrete is still not effectively utilized. Meanwhile, cement production contributes approximately 5–8% of global anthropogenic CO_2_ emissions, creating significant pressure for carbon reduction in the construction sector [5,6]. To address these issues, increasing attention has been paid to the development of sustainable construction materials based on waste resources, in line with the concept of a circular economy [7].

Among various recycling strategies, the incorporation of recycled concrete waste into cement-based materials has been widely investigated [8]. In particular, waste concrete can be processed into recycled concrete powder (RCP) and reused as a supplementary material in cement systems. Existing studies have shown that RCP mainly consists of residual cement paste (including C-S-H gels and Ca(OH)_2_) and fine aggregates, which provide limited intrinsic reactivity but can still participate in hydration through filler effects and partial pozzolanic reactions. The incorporation of RCP has been reported to influence the workability, setting behavior, and mechanical properties of cementitious materials. Zhang et al. [9] reported that workability reached an optimum at 10% RCP replacement, while higher contents led to a reduction due to increased water demand from the fine and angular particles. Likes et al. [10] found that the addition of 20% RCP led to a reduction in early compressive strength (14% at 7 d) but gradually recovered at 28 d, reaching comparable strength to the control at 56 d. However, at higher replacement levels (>20%), a more pronounced strength loss is commonly observed. For example, Wu et al. [11] and Yang et al. [12] found that replacing 30% of cement resulted in an approximately 30% reduction in compressive strength, mainly due to clinker dilution and the low reactivity of RCP.

To improve the reactivity of RCP, several modification methods have been explored, including calcination [13], grinding [14], and carbonation [15,16]. Among these approaches, carbonation has attracted considerable attention because it not only enhances the properties of RCP but also contributes to CO_2_ sequestration. The carbonation of RCP typically falls into two categories: aqueous carbonation [17] and gas–solid carbonation [18]. Between these two approaches, the aqueous route is widely regarded as more effective and offers greater operational flexibility [19,20]. During aqueous carbonation, the residual cementitious fraction within RCP can be rapidly transformed into CaCO_3_ and amorphous products, primarily silica gel, within a short reaction period. According to Shen et al. [21], the formation of silica gel takes place within the first 30 min of aqueous carbonation. Yang et al. [22] further observed that CaCO_3_ and silica gel are evenly deposited across the particle surfaces of RCP, resulting in a marked decrease in pore volume. Moreover, earlier investigations [23,24] have revealed that the resulting calcite silica gel composite possesses relatively high pozzolanic reactivity. Owing to these favorable properties, carbonated RCP has been explored as a sustainable alternative supplementary cementitious material. In particular, Zhang et al. [25] demonstrated that carbonated RCP can accelerate cement hydration through both nucleation effects and the physical filling provided by fine CaCO_3_ particles. Similarly, Lu et al. [26] reported that its incorporation can densify the microstructure and improve compressive strength due to the combined filler and reactive effects.

Although carbonation treatment improves the performance of RCP, its application in blended cement systems remains insufficiently understood, particularly in ternary systems incorporating supplementary cementitious materials such as fly ash. Most existing studies have primarily focused on binary systems and mechanical properties, while the combined effects of carbonated RCP (CRCP) on fresh behavior, hydration kinetics, microstructure evolution, and shrinkage characteristics have not been systematically clarified. In addition, the influence of replacement level on the balance between filler effect, nucleation effect, and potential reactivity of CRCP still requires further investigation.

In this study, CRCP was used as a partial replacement for cement in Portland cement–fly ash (OPC-FA) systems at substitution levels of 4%, 8%, 12%, and 16%. The study initially assessed how carbonated RCP influences workability and setting behavior. Subsequently, a detailed analysis of hydration kinetics was performed using thermogravimetric analysis (TG), X-ray diffraction (XRD), and isothermal calorimetry. In addition, both autogenous and drying shrinkage were measured to evaluate volumetric stability. To further understand the microstructural evolution, mercury intrusion porosimetry (MIP) and nanoindentation tests were conducted to examine pore characteristics and local mechanical properties. By integrating these multi-scale measurements, this work aims to offer a holistic understanding of the functional role of carbonated RCP in blended cement systems and to explore its feasibility as an eco-friendly cement replacement.

## 2. Materials and Methods

### 2.1. Materials

**Figure 1:**
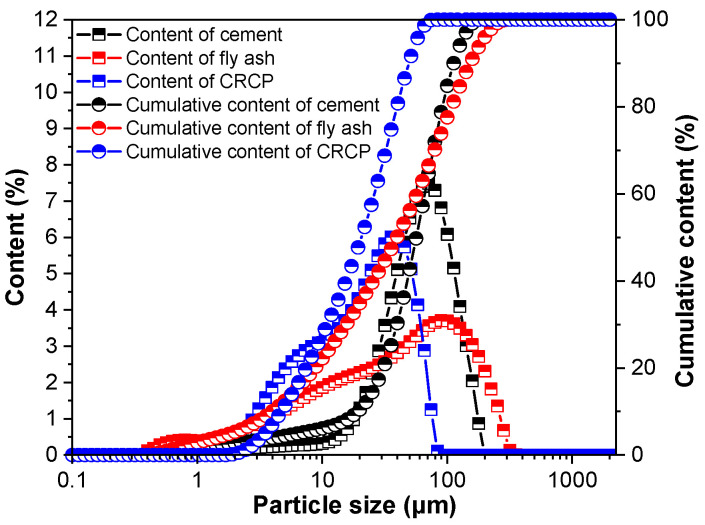
Particle size distributions of cement, fly ash and CRCP.

In this study, Ordinary Portland cement (Grade P·O·42.5) produced by Huaxin Cement Co., Ltd. (Huangshi, Hubei, China) was utilized, exhibiting a BET-specific surface area of 1.436 m^2^/g. Natural river sand with a fineness modulus of 2.55 served as the fine aggregate. A low-calcium Class F fly ash (FA) was adopted as a supplementary cementitious material, and municipal tap water was used for all mixture preparations. The use of carbonated recycled concrete powder (CRCP) was prepared by carbonizing recycled concrete powder (RCP). The experimental procedures followed the steps outlined in Section 2.2. Chemical compositions of the starting materials were determined via X-ray fluorescence (XRF) spectrometry, and the corresponding outcomes are presented in Table 1. Particle size distributions of cement, fly ash, and CRCP were measured using a dynamic light scattering (DLS) particle size analyzer, and the resultant curves are shown in Figure 1.

### 2.2. Preparation Process of CRCP

For the carbonation treatment, RCP was first dispersed in water at a solid-to-liquid mass ratio of 1:10 to form a homogeneous suspension [27,28]. The mixture was allowed to rest for 10 min before carbonation. Subsequently, CO_2_ gas was continuously introduced into the suspension through a perforated tube positioned at the bottom of the vessel, with a flow rate of 1.5 L/min. To enhance gas utilization, the reactor was sealed with a plastic film during the reaction. Meanwhile, the suspension was mechanically stirred at 300 rpm to maintain uniform dispersion. The carbonation process was sustained for 6 h to ensure sufficient reaction. Afterwards, the carbonated slurry was separated using membrane filtration (200 nm), and the obtained carbonated RCP (CRCP) was dried under vacuum at 40 °C until a constant mass was reached.

**Figure 2:**
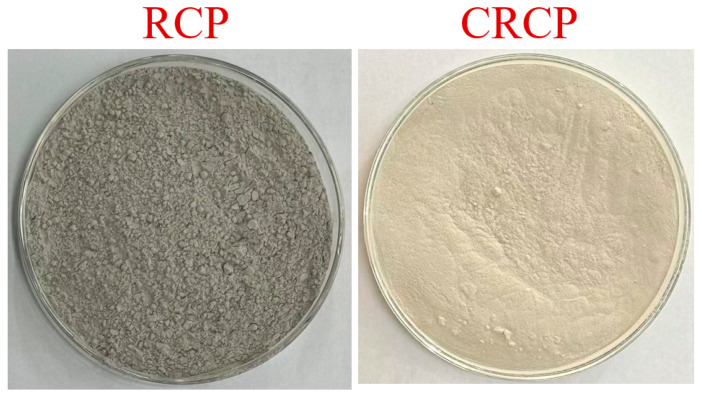
Visual appearance of RCP and CRCP.

**Figure 3:**
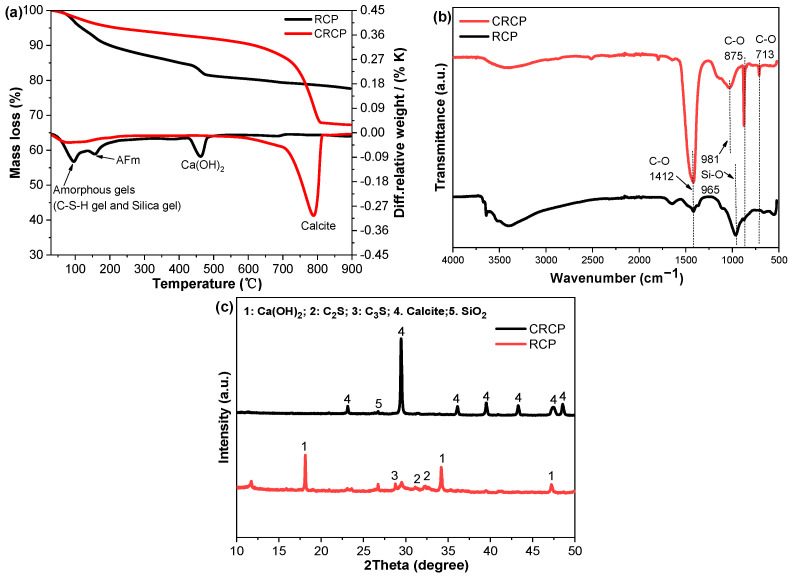
(**a**) TG-DTG curves, (**b**) FTIR spectra, and (**c**) XRD patterns of RCP and CRCP.

The visual appearance of RCP and CRCP is presented in Figure 2. Thermogravimetric (TG) analysis was conducted to evaluate the carbonation degree, as shown in Figure 3a. The results indicate that CRCP was nearly fully carbonated, as evidenced by a dominant decomposed peak in the range of 700–800 °C, which is associated with the decomposition of CaCO_3_. In addition, Fourier transform infrared (FTIR) spectra exhibit characteristic C–O stretching vibrations of carbonate groups (Figure 3b), further confirming the formation of calcium carbonate after carbonation. At the same time, from the X-ray diffraction (XRD) patterns, it can be seen that after carbonization treatment of RCP, the peak corresponding to Ca(OH)_2_ gradually disappeared, while the peak corresponding to calcium carbonate significantly increased and the peak value was significantly enhanced, as shown in Figure 3c. This suggests that CRCP contains a significant amount of CaCO_3_.

### 2.3. Sample Preparation

This study employed CRCP as a partial substitute for cement, with replacement content set at 4%, 8%, 12%, and 16% relative to the cement mass. A constant water-to-binder (w/b) ratio of 0.34 was maintained across all mixtures. The complete mixture proportions are summarized in Table 2. Prior to mixing, cement, FA, and CRCP were dry-blended to ensure a uniform distribution of the solid constituents. Subsequently, the mixing procedure was carried out in accordance with the Chinese standard for cement paste preparation. Specifically, the pre-mixed binder was first mixed with water at low speed, followed by high-speed mixing to obtain a homogeneous ternary cement paste. For mortar preparation, standard sand was gradually added to the paste after mixing at low speed, and the mixture was further mixed until a uniform mortar was obtained. Upon completion of mixing, the freshly prepared paste or mortar was placed into molds and subjected to vibration to remove any trapped air bubbles. The molded specimens were subsequently sealed to avoid moisture evaporation and then maintained under standard curing conditions (20 °C, relative humidity ≥ 95%) until the predetermined testing ages.

### 2.4. Methods

#### 2.4.1. Fresh Properties

The setting time of the cement pastes was measured following ASTM C191 [29] using a Vicat needle apparatus, with all specimens placed under standard curing conditions (23 ± 2 °C, relative humidity not below 95%). For flowability assessment, fresh mortar was loaded into a mold designed for flow table testing. After filling and leveling the top surface, the mold was raised vertically without any lateral displacement.

The flowability of the samples was tested in accordance with ASTM C1437 [30]. The flow table was then raised and dropped 25 times at a consistent rate, causing the mortar to spread into a circular patty. The fluidity value was defined as the average of two perpendicular diameter measurements of the final spread mortar, recorded immediately after the last jolt. This test was conducted in triplicate to ensure reproducibility.

#### 2.4.2. Strength

In accordance with the Chinese Standard GB/T 17671–2021 [31], the compressive and flexural strengths of the mortar specimens were measured. The test specimens, shaped as 40 mm × 40 mm × 160 mm prisms, underwent standard curing for periods of 3, 7, and 28 days before being subjected to mechanical testing. Flexural strength tests were conducted first, after which the resulting two halves from each broken prism were collected and used for compressive strength measurements. All mechanical tests were performed on an automatic testing machine (WHY-300, Shanghai Hualong Test Instruments Co., Ltd., Shanghai, China). During the mechanical tests, the applied loading speeds were maintained at 60 N/s for flexural strength measurement and 2.3 kN/s for compressive strength measurement, respectively. The final flexural strength values are reported as the mean of three replicate tests, while the compressive strength results represent the average of six measurements.

#### 2.4.3. Hydration Heat

The progression of cement hydration was monitored under isothermal conditions maintained at 25 °C using an eight-channel isothermal calorimeter. At precisely three minutes after the initial contact between cement and water, each freshly mixed sample was sealed into a glass ampoule to prevent moisture loss or carbonation. The sealed ampoules were then immediately inserted into the calorimeter, which continuously recorded the rate of heat liberation throughout the early and subsequent hydration stages. This setup allowed for high-sensitivity detection of thermal evolution, providing reliable data on hydration kinetics without disturbing the sample environment.

#### 2.4.4. Hydration Products

The crystalline phases of the hydrated cement pastes were identified using X-ray diffraction (XRD; D8 Advance, Bruker Corporation, Karlsruhe, Germany). To halt further hydration prior to analysis, the fragmented paste samples were immersed in isopropanol for 48 h, followed by vacuum drying and subsequent pulverization into a homogeneous fine powder. Diffraction patterns were recorded across a 2θ range spanning from 5° to 50°. In addition to the phase identification, a supplementary thermogravimetric analysis (TGA) was conducted on aliquots of the same powdered material to enable quantitative evaluation of the hydration products. In each TGA measurement, the specimen was heated from 40 °C up to 800 °C, and the corresponding weight loss was recorded continuously throughout the process. This allowed for the characterization of the thermal decomposition behavior exhibited by the different phases present in the sample.

#### 2.4.5. Shrinkage Behavior Test

For drying shrinkage assessment, prismatic specimens were first subjected to initial curing until saturation, after which their initial length and mass were recorded as reference values. These samples were then placed in a climate-controlled chamber maintained at 25 °C and 50% relative humidity for a duration of 28 days. Throughout this period, both mass and length measurements were taken at regular intervals to capture dimensional variations. Autogenous shrinkage was evaluated following the corrugated tube method prescribed in ASTM C1698 [32]. Specifically, the freshly mixed paste was poured into a polyethylene tube (425 mm in length, 29 mm in inner diameter) with corrugated inner walls. The tube was subsequently connected to a dilatometer equipped with a high-precision linear variable differential transformer (LVDT) gauge (GT-2000, Keyence Corporation, Osaka, Japan). Starting from the moment when the final setting time of the mixture was identified, length change readings were automatically logged at one-minute intervals, enabling continuous monitoring of the early-age autogenous deformation.

#### 2.4.6. Internal Relative Humidity (IRH)

To evaluate the internal curing condition of the cement-based materials, the IRH was continuously monitored using 100 mm cubic specimens placed in a climate-controlled chamber set at 20 ± 3 °C and 95% RH. A digital resistive humidity sensor (HIH-4000, Honeywell International Inc., Charlotte, NC, USA), connected to a data logging system (CR1000, Campbell Scientific, Inc., Logan, UT, USA), was embedded into each specimen for automated data acquisition. This sensor setup offers reliable accuracy, with ±0.5 °C for temperature and ±3% for relative humidity. 

#### 2.4.7. Surface Tension

To obtain the pore solution for analysis, the freshly mixed cement paste was allowed to stand briefly for solid particles to settle, after which the supernatant liquid was separated by vacuum filtration using a 0.45 μm membrane filter (MF-Millipore™, Merck KGaA, Darmstadt, Germany). After extraction, the pore solution was tested for surface tension using an automated tensiometer (K100, KRÜSS GmbH, Hamburg, Germany) that operates based on the Wilhelmy plate principle. To ensure reproducibility, three consecutive measurements were performed on each solution sample, and the final surface tension value was calculated as the arithmetic mean of the three individual readings, with a reported precision of ±0.1 mN/m.

#### 2.4.8. Microstructure Characteristics Test

Mercury Intrusion Porosimetry (MIP; AutoPore V 9620, Micromeritics Instrument Corporation, Norcross, GA, USA) was employed to characterize the pore structure of mortar specimens after 28 days of curing. Small fragments, each measuring no more than 5 mm in its smallest dimension, were collected from the samples previously used for mechanical testing. To stop any ongoing hydration, these fragments were submerged in isopropanol for 48 h and subsequently dried under vacuum at 40 °C until a stable weight was reached before MIP analysis.

The micromechanical properties of the cementitious samples were investigated using a nanoindentation system (TI 950 Premier Nanoindenter, Bruker Corporation, Billerica, MA, USA). For this purpose, the specimens were embedded in epoxy resin and subsequently subjected to a sequential grinding and polishing procedure, employing progressively finer silicon carbide abrasive papers (down to 4000 grit) and final polishing with 0.25 μm diamond suspensions to achieve a mirror-like surface finish. A predefined grid pattern of indents was applied with a uniform spacing of 50 μm between adjacent indentation points. At each test location, a controlled loading–unloading cycle was performed, consisting of a 10 s loading segment to a peak load of 2 mN, a 5 s hold period at the maximum load, and a 10 s unloading segment. Based on the acquired load–displacement curves, the elastic modulus was derived using the analytical procedure proposed by Oliver and Pharr [33], with a Poisson’s ratio of 0.2 assumed for the tested material.

## 3. Results and Discussions

### 3.1. Setting Time and Fluidity

**Figure 4:**
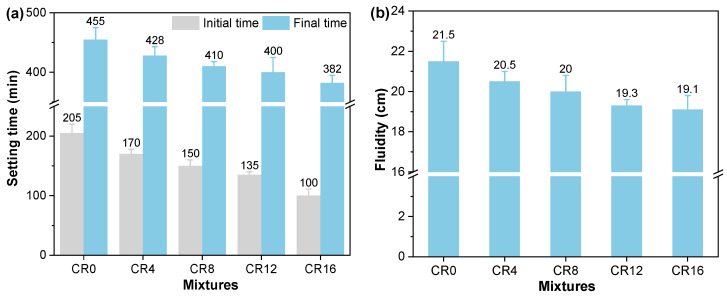
Effects of CRCP on the (**a**) setting time and (**b**) flowability of the ternary mixtures.

The effects of CRCP on the setting time and flowability of the mixtures are presented in Figure 4. As the CRCP replacement level increased, both the initial and final setting times gradually decreased. Compared with the control mixture (CR0), the initial setting time decreased by 17.1%, 26.8%, 34.1%, and 51.2% at replacement levels of 4%, 8%, 12%, and 16%, respectively, while the final setting time showed a similar reduction of 5.9%, 9.9%, 12.1%, and 16.0%. The reduction in setting time can be attributed to the nucleation effect of fine CaCO_3_ particles formed during carbonation [34], which accelerates early hydration reactions. The finer particle characteristics of CRCP lead to a larger specific surface area, which supplies additional sites for the attachment and growth of hydration products. Consequently, the setting process was accelerated.

In addition, a similar decreasing trend was observed in flowability. As shown in Figure 4b, the flow diameter decreased progressively with increasing CRCP content, with reductions of 4.6%, 7.0%, 10.2%, and 11.2% compared to CR0. This reduction could be partly attributed to the finer particle size of CRCP relative to cement and fly ash, which increases the specific surface area and thus the water demand of the system [35]. In addition, the presence of carbonated phases on the particle surface may enhance interparticle interactions, further limiting flowability. Similar observations have been widely reported in previous studies on recycled powders and carbonated materials [36,37].

### 3.2. Hydration Process

The heat evolution of cement-based materials typically exhibits several characteristic stages, including the initial dissolution peak, induction period, acceleration period associated with the main hydration peak, and the subsequent deceleration stage [38,39]. The prominent exothermic peak is typically associated with the accelerated generation of hydration products, which primarily stems from the reaction of C_3_S as well as the precipitation of C–S–H and Ca(OH)_2_ [40,41].

**Figure 5:**
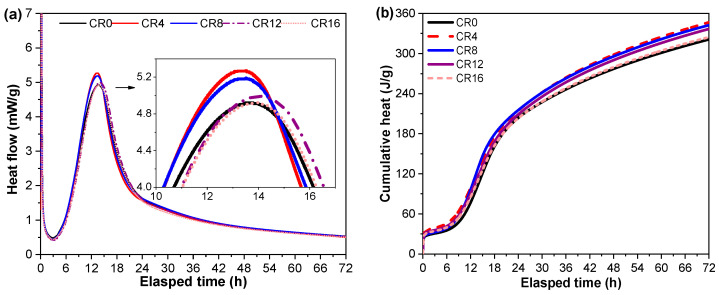
Effects of CRCP on the hydration process of the ternary mixtures: (**a**) heat flow; (**b**) cumulative heat.

The heat flow curves of the ternary mixtures are shown in Figure 5a. The incorporation of CRCP shows a limited influence on the induction period, indicating that the early dissolution process is not significantly affected. However, slight shifts in the main hydration peak can be observed. At low replacement levels (4% and 8%), the main exothermic peak appears slightly earlier compared to CR0, accompanied by an increase in peak intensity, particularly at 4%, where the highest heat flow is observed. This phenomenon is explained by the nucleation effect provided by the fine CaCO_3_ particles present in CRCP [42,43]. These particles serve as nucleation sites that facilitate the deposition of hydration products and accelerate the reaction of the cement clinker phases. In addition, the filler effect may improve particle packing, facilitating ion diffusion and early hydration reactions [44]. However, as the replacement level further increases, the peak intensity gradually decreases. This reduction is mainly associated with the dilution effect [45], where the decrease in clinker content limits the availability of reactive phases, thereby reducing the corresponding heat flow.

Regarding cumulative heat (Figure 5b), the incorporation of CRCP slightly increases the total heat release within 72 h compared to CR0. This may be related to the combined effects of accelerated early hydration and the contribution of fine particles to the overall reaction process [46,47].

### 3.3. Hydration Product

To further clarify the influence of CRCP on the hydration of ternary mixtures, the evolution of hydration products was examined. Since calorimetry reflects only reaction kinetics, XRD and TG are used to identify phase composition and quantify hydration products, which are closely related to strength and microstructure [48,49].

**Figure 6:**
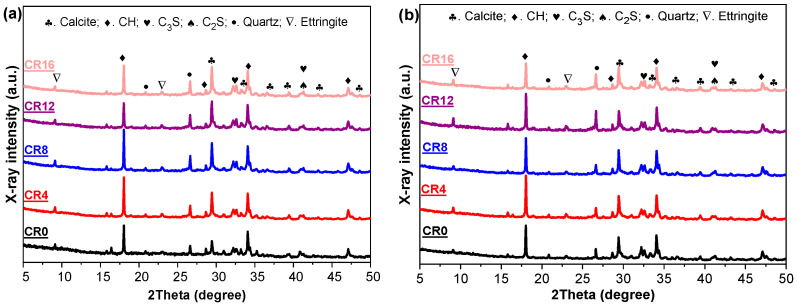
XRD patterns of the ternary mixtures at (**a**) 3 d and (**b**) 28 d.

The XRD patterns of the samples at 3 d and 28 d are presented in Figure 6. The main crystalline phases identified include calcite, portlandite (CH), C_3_S, C_2_S, quartz, and ettringite (AFt). At 3 d, compared with the CR0, the incorporation of CRCP leads to the appearance of distinct diffraction peaks about calcite, confirming the presence of carbonation products. In addition, the intensity of the CH peaks increases at low replacement levels, indicating an accelerated hydration process. However, as the CRCP content further increases to 12% and 16%, the peak intensity of CH decreases. This trend is consistent with the results of isothermal calorimetry, where an enhancement in early hydration is observed at low dosages, followed by a reduction at higher replacement levels. At 28 d, the differences in peak intensities among the ternary mixtures become less pronounced, suggesting that the influence of CRCP on hydration products tends to diminish over time. The CH peaks of all mixtures show comparable intensities, indicating that the long-term hydration degree gradually remains comparable. Although the presence of carbonates may promote the formation of carboaluminate phases, no distinct diffraction peaks attributable to such phases are identified, possibly due to their low content and overlap with other hydration products.

**Figure 7:**
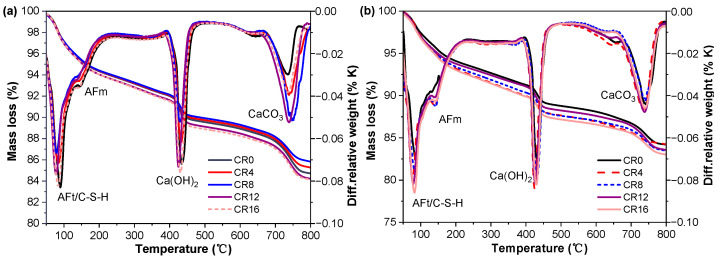
TG and DTG curves of the samples at different curing ages: (**a**) 3 d; (**b**) 28 d.

Given that XRD is primarily effective for detecting crystalline phases and exhibits limited sensitivity toward amorphous hydration products such as C-S-H and AFm [50,51], TG analysis was additionally performed to better characterize the hydration products. The resulting TG and DTG curves are shown in Figure 7. From the DTG profiles, several distinct mass loss regions can be distinguished, which correspond to the thermal decomposition of various phases: ettringite and C-S-H decompose between approximately 80 and 105 °C, AFm between 180 and 200 °C, Ca(OH)_2_ between 400 and 500 °C, and CaCO_3_ between 650 and 800 °C [38,52].

At 3 d, the incorporation of CRCP leads to a reduction in the mass loss associated with AFt/C–S–H compared to CR0, indicating a lower amount of early hydration products. In contrast, the mass loss attributed to CH increases when CRCP is added, which is consistent with the XRD results and suggests an accelerated early hydration process. In addition, a pronounced mass loss peak related to CaCO_3_ is observed in CRCP-containing mixtures, confirming the presence of carbonation products. At 28 d, a different trend is observed. The mass loss associated with AFt/C–S–H increases progressively with increasing CRCP content, indicating enhanced formation of hydration products at later ages. A similar increasing trend is also observed for AFm and CH. However, the mass loss corresponding to CaCO_3_ shows limited variation among different mixtures, which may be attributed to partial carbonation of all samples during curing in air [52]. The increase in C–S–H at 28 d can be attributed to the combined effects of filler and nucleation provided by fine CaCO_3_ particles [53], which promote continued hydration of cement and fly ash. In addition, the improved particle packing may facilitate ion transport, further enhancing the formation of hydration products over time [54].

### 3.4. Shrinkage Behavior

**Figure 8:**
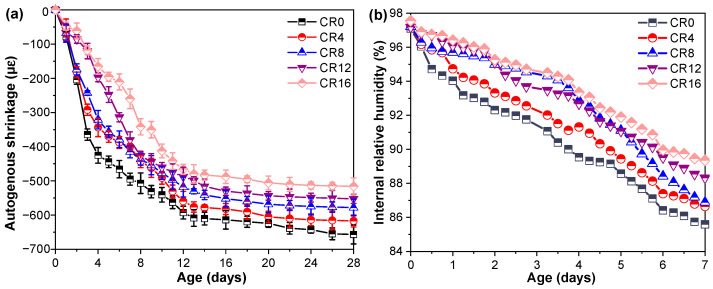
(**a**) Autogenous shrinkage and (**b**) Internal relative humidity of samples.

Shrinkage is a key factor affecting the durability and dimensional stability of ternary mixtures [55,56]. The incorporation of CRCP could influence shrinkage by modifying internal moisture conditions and capillary stress. Therefore, the effect of CRCP on autogenous and drying shrinkage was investigated to clarify its role in volume stability. Autogenous shrinkage results of the ternary mixtures are presented in Figure 8a. It can be observed that the incorporation of CRCP significantly reduces the autogenous shrinkage of the ternary mixtures. Specifically, compared with the CR0, the 28 d autogenous shrinkage decreased by 6.0%, 12.0%, 15.9%, and 21.4% at CRCP replacement levels of 4%, 8%, 12%, and 16%, respectively. To further understand this behavior, the IRH of the mortars was monitored, as shown in Figure 8b. The results indicate that the IRH at 7 d increases with increasing CRCP content, with increments of 1.2%, 1.5%, 3.2%, and 4.4% compared to CR0.

The reduction in autogenous shrinkage can be attributed to the mitigation of self-desiccation within the system. Although CRCP particles may exhibit a certain water absorption capacity [39,57], the presence of carbonated products (e.g., CaCO_3_ and silica gel) and the modified pore structure can help retain moisture within the matrix. The increased internal RH suggests that water consumption during hydration is effectively alleviated, thereby reducing capillary stress development. In addition, the filler effect of fine CRCP particles may refine the pore structure and interrupt the connectivity of capillary pores, further mitigating shrinkage. Similar trends have been reported in previous studies on fine mineral additions and carbonated materials [58,59].

**Figure 9:**
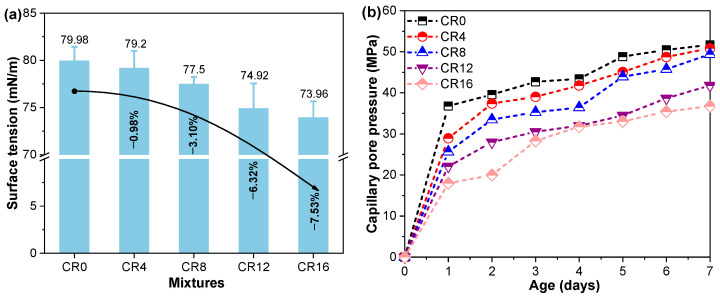
(**a**) Surface tension of pore solution; (**b**) Calculated capillary pressure of samples.

The surface tension of sample was further measured as a means of verifying the above hypothesis, and the outcomes are presented in Figure 9a. It can be observed that the surface tension decreases progressively with increasing CRCP content, with reductions of 0.98%, 3.10%, 6.32%, and 7.53% compared to CR0.

Using the measured internal relative humidity and surface tension values, the capillary pressure was determined through Equations (1)–(5), with the results presented in Figure 9b. It should be noted that the calculated capillary pressure is intended for qualitative comparison and trend analysis, rather than a direct experimental measurement. This calculation follows the Kelvin–Laplace formulation, in which *γ* (N/m) stands for surface tension, *θ*(°) for contact angle, and *r* (m) for the Kelvin radius. The term *V_W_* represents the molar volume of water, *R* denotes the universal gas constant (8.314 J/(mol·K)), and *T* indicates the absolute temperature (293.15 K). *RH_K_* represents the relative humidity associated with meniscus curvature, while RH refers to the measured internal relative humidity of the pore system. *RH_S_* accounts for the reduction in relative humidity due to dissolved ions, which is calculated according to Raoult’s law (Equation (4)), where *n_H_*_2*O*_ and *n_solution_* denote the molar amounts of water and solution, respectively. According to Lura [60], the influence of ions on *V_W_* can be neglected; therefore, the molar volume of pure water (18.02 × 10^−6^ m^3^/mol) was adopted in this study.

(1) $$\sigma_{cap}=\frac{2\gamma cos\theta }{r}$$

(2) $$r=\frac{2\gamma V_{w}cos\theta }{ln(RH_{K})RT}$$

(3) $${RH}_{K}=\frac{RH}{{RH}_{S}}$$

(4) $${RH}_{S}=\frac{n_{H_{2}O}}{n_{solution}}$$

(5) $$\sigma_{cap}=-\frac{ln(RH_{K})RT}{V_{W}}$$

As expected, the incorporation of CRCP leads to a noticeable reduction in capillary pressure. Specifically, compared with CR0, the capillary pressure decreases by 1.6%, 4.4%, 19.1%, and 28.9% at replacement levels of 4%, 8%, 12%, and 16%, respectively. This reduction can be attributed to the combined effects of lower surface tension and higher internal relative humidity, both of which contribute to alleviating capillary stress. Consequently, the decrease in capillary pressure provides a direct explanation for the observed mitigation of autogenous shrinkage in CRCP-containing ternary mixtures.

**Figure 10:**
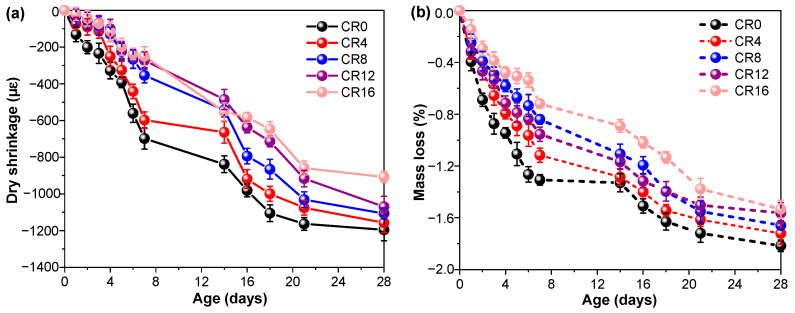
(**a**) Drying shrinkage of samples and (**b**) mass loss of samples during drying.

In addition to autogenous shrinkage, drying shrinkage was also evaluated to further assess the dimensional stability of the ternary mixtures. As shown in Figure 10a, a similar trend to that of autogenous shrinkage is observed. The incorporation of CRCP leads to a noticeable reduction in drying shrinkage. Specifically, compared with the CR0, the drying shrinkage at 28 d decreases by 3.2%, 7.4%, 10.5%, and 24.1% at CRCP replacement levels of 4%, 8%, 12%, and 16%, respectively. To further understand this behavior, the mass loss of the specimens was monitored during the drying process, as presented in Figure 10b. It can be observed that, although the incorporation of CRCP reduces the mass loss, the extent of reduction gradually diminishes with increasing CRCP content. The mass loss is primarily associated with the evaporation of pore water, and thus reflects the moisture transport and retention characteristics of the material [15,61]. The reduced drying shrinkage can be attributed to the lower moisture loss and the modified pore structure induced by CRCP incorporation. The presence of fine particles and carbonation products (e.g., CaCO_3_) contributes to pore refinement and reduced connectivity of capillary pores, thereby limiting water evaporation [62]. In addition, the decreased surface tension and capillary pressure, as discussed previously, further alleviate shrinkage of samples.

### 3.5. Mechanical Properties

**Figure 11:**
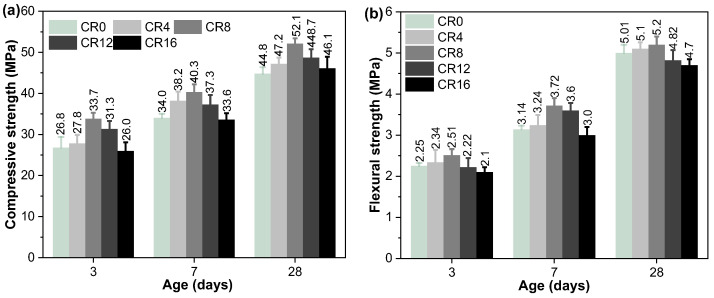
(**a**) Compressive strength and (**b**) flexural strength of hardened mortars.

The evolution of compressive strength for hardened mortars at different curing ages is displayed in Figure 11a. When cured for 3 days, the strength exhibits a trend of increasing initially and then decreasing with higher CRCP content. CR8 exhibits the highest compressive strength among all tested mixtures, which was 26.1% higher than that observed for CR0. Although further increasing the CRCP content leads to a reduction in strength, the mixture with 12% CRCP still retains a compressive strength higher than that of CR0. A further increase in CRCP content to 16% results in a compressive strength lower than that of CR0. The reduction in compressive strength at higher replacement levels can be mainly attributed to the dilution effect [37], where the decreased clinker content limits the formation of hydration products. In addition, excessive incorporation of CRCP may introduce more porous or less reactive phases, which adversely affect the mechanical performance.

A similar trend is observed at 7 d, where CR8 still exhibits the highest compressive strength. As the hydration time extends to 28 d, the compressive strength continues to show a comparable trend, with CR8 achieving the highest value. Notably, even at a replacement level of 16%, the compressive strength surpasses that of CR0. This improvement at later ages can be associated with the continued formation of C–S–H, as evidenced by TG results. Furthermore, the active silica gel produced during the carbonation process can facilitate the generation of supplementary C–S–H phases [63,64], which positively affects the compressive strength.

The flexural strength data for hardened mortars are presented in Figure 11b. A similar pattern to the compressive strength can be observed. Specifically, at 3 days, raising the CRCP level leads to an initial enhancement followed by a reduction in flexural strength, with the highest value recorded for CR8. This enhancement is ascribed to the combined effects of particle filling by fine CRCP particles and the nucleation effect of CaCO_3_, which together accelerate early-age hydration and produce a more compact microstructure [64]. At 7 d, this trend remains consistent, indicating that moderate incorporation of CRCP is beneficial to early-age mechanical performance. At 28 d, although the overall trend is still maintained, the flexural strength of mixtures with higher CRCP contents (12% and 16%) is slightly lower than that of CR0. This reduction may be related to the dilution of clinker phases at higher replacement levels, as well as the relatively weaker bonding at the interface between CRCP particles and the cement matrix [37], which makes the material more susceptible to crack propagation under bending.

### 3.6. Pore Structure

**Figure 12:**
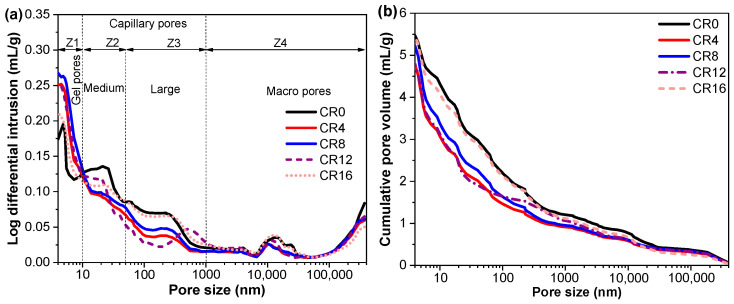
MIP results of 28 d samples: (**a**) pore size distributions; (**b**) cumulative pore volumes.

MIP was employed to characterize the pore structure of the mortars, and the resulting pore size distribution is presented in Figure 12. Following the classification schemes proposed in earlier investigations [65,66], the pore network can be divided into four categories: gel pores (designated as Z1, with diameters below 10 nm), medium capillary pores (Z2, ranging from 10 to 50 nm), large capillary pores (Z3, spanning 50–1000 nm), and macropores (Z4, exceeding 1000 nm). Among these, gel pores are typically linked to C-S-H formation and contribute favorably to mechanical strength [67]. Capillary pores in the 10–1000 nm range play a crucial role in shrinkage behavior, as they govern the development of capillary stress during moisture evaporation [68]. Macropores (larger than 1000 nm) are primarily responsible for strength reduction [68], since they act as weak regions that facilitate crack initiation and propagation under applied loads.

To further quantify the pore structure, the cumulative pore volumes within each range are summarized in Table 3. It can be observed that the incorporation of CRCP significantly affects the pore distribution. The volume of gel pores (Z1) increases noticeably with the addition of CRCP, reaching the highest values at CR8, indicating enhanced formation of fine pores. In contrast, the volume of large capillary pores and macro pores (Z3 and Z4), which are generally considered harmful, decreases when the CRCP is incorporated, suggesting a refinement of the pore structure. Specifically, the combined volume of harmful pores (Z3 + Z4) shows a clear reduction compared with CR0. The total volume of Z3 + Z4 decreases by 12.1%, 21.8%, 6.4%, and 3.5% for CR4, CR8, CR12, and CR16, respectively, indicating a substantial reduction in pores that could be primarily responsible for capillary stress development. This reduction in harmful pore volume is consistent with the compressive strength results, where mixtures with lower Z3 + Z4 content exhibit higher strength. Meanwhile, although the fraction of gel pores increases, these pores could contain strongly adsorbed water and contribute less to capillary pressure, suggesting that the overall shrinkage behavior could be governed by the reduction in larger capillary pores. These findings further confirm that the improvement in mechanical performance is closely associated with the refinement of pore structure.

In addition, the porosity and pore structure parameters are listed in Table 4. The total porosity shows a decreasing trend at lower CRCP contents and reaches the minimum at CR8, followed by an increase at higher replacement levels. This trend is also consistent with the compressive strength results, where CR8 exhibits the highest strength. The reduction in porosity and refinement of pore size distribution contribute to a denser microstructure, thereby enhancing mechanical performance. The reduced porosity together with the shift from larger capillary pores to finer pores leads to a denser and more homogeneous microstructure, which is beneficial for both mechanical performance and dimensional stability. Conversely, the increase in porosity at higher replacement levels can be attributed to the dilution effect and reduced formation of hydration products, which negatively affect strength development [65,66].

### 3.7. Nanoindentation Analysis

**Figure 13:**
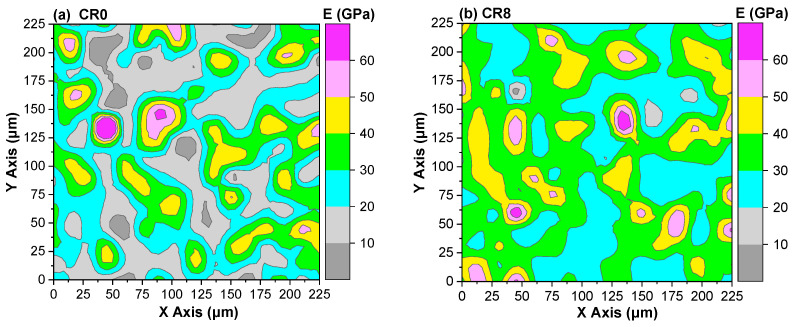
Elastic modulus mapping of mortars at 28 d: (**a**) CR0; (**b**) CR8.

**Figure 14:**
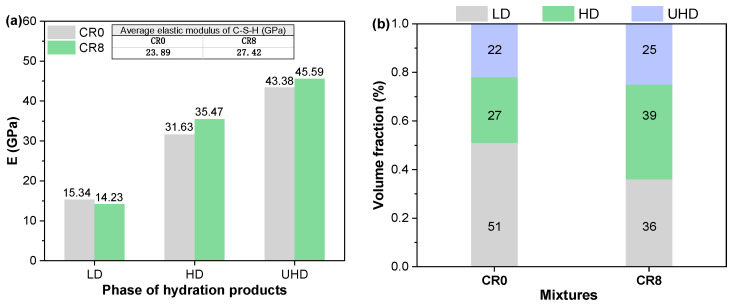
Statistical deconvolution of elastic modulus (**a**) and volume fractions (**b**) of LD C–S–H, HD C–S–H, and UHD C–S–H for CR0 and CR8 at 28 d.

To further clarify the influence of CRCP on the micromechanical properties of the hardened samples, nanoindentation tests were conducted on CR0 and CR8, because CR8 shows the highest compressive strength. The elastic modulus mappings and corresponding deconvolution results at 28 d are shown in Figure 13 and Figure 14, respectively. Based on the elastic modulus distribution, different regions can be distinguished. Regions with low elastic modulus (typically <20 GPa) are generally associated with pores, defects, or loosely packed hydration products [65], whereas intermediate modulus ranges (20–30 GPa) correspond to low-density C–S–H (LD C–S–H) [66]. Higher modulus regions (30–50 GPa and above) are mainly attributed to high-density (HD) and ultra-high-density (UHD) C–S–H, which are responsible for the mechanical strength of the matrix [66]. As shown in Figure 13, CR0 exhibits a relatively larger fraction of low-modulus regions (<20 GPa), indicating the presence of more porous or weak zones. In contrast, these regions are significantly reduced in CR8, while the proportion of high-modulus areas (>30 GPa) is markedly increased, suggesting the formation of a denser and mechanically stronger microstructure.

Quantitative support for these observations was obtained through statistical deconvolution of the nanoindentation data, as presented in Figure 14. In accordance with previously established classifications [65,66], the hydration products were differentiated into LD C-S-H, HD C-S-H, and UHD C-S-H, and their respective volume fractions were calculated. The results demonstrate that the CR8 mixture contains a significantly lower volume fraction of LD C-S-H compared to CR0. Conversely, the proportions of HD C-S-H and UHD C-S-H in CR8 are substantially higher, exhibiting increases of 12.0% and 3.0%, respectively. In addition, the elastic moduli of both HD and UHD C-S-H in CR8 are higher than those in CR0, suggesting an overall improvement in phase stiffness. The average elastic modulus was further calculated, yielding values of 23.89 GPa for CR0 and 27.42 GPa for CR8, corresponding to an increase of approximately 14.8%. This enhancement in micromechanical properties is consistent with the MIP and TG results, which indicate a refined pore structure and increased formation of C-S-H. The incorporation of CRCP, particularly at an optimal dosage (CR8), promotes the formation of denser hydration products and reduces weak phases, thereby improving the strength of hardened mortars.

## 4. Conclusions

This study examines the use of carbonated recycled concrete powder (CRCP) as a partial cement replacement (4–16%) in Portland cement–fly ash (OPC-FA) systems, with emphasis on its influence on hydration, shrinkage, pore structure, and mechanical performance.

The incorporation of CRCP reduces flowability and shortens setting time, while also modifying the hydration process, with a slightly advanced and enhanced main hydration peak at 4–8% replacement, accompanied by higher CH at early ages and increased C–S–H formation at later stages. Furthermore, the incorporation of CRCP markedly reduces both autogenous and drying shrinkage, with reductions of 6.0–21.4% and 3.2–24.1%, respectively, which is attributed to improved internal moisture conditions and reduced capillary stress. Meanwhile, the pore structure of samples is refined, with an increased fraction of gel pores and a reduction in larger pores (Z3 + Z4). As a result, both compressive and flexural strengths of hardened samples reach their highest values at 8% replacement, exceeding the reference by 16.3% and 4.0% at 28 d, respectively. Although higher CRCP dosages lead to further shrinkage reduction, they are accompanied by a decrease in mechanical strength due to dilution effects and increased porosity, indicating a trade-off between dimensional stability and mechanical performance. At the microscale, this is reflected by a reduction in low-modulus regions (<20 GPa), an increase in high-modulus regions (>30 GPa), a shift from LD C–S–H to HD and UHD C–S–H, and an increase in average elastic modulus from 23.89 GPa to 27.42 GPa, indicating the formation of a stiffer and more compact hydration structure. Therefore, an optimal replacement level of 8% is identified to achieve a balanced improvement in both strength and shrinkage performance. These findings demonstrate that CRCP can be effectively utilized as a cement-replacing material to improve both dimensional stability and mechanical performance, while providing a viable route for the value-added utilization of recycled concrete powder.

This study mainly focuses on the effects of CRCP on hydration behavior, shrinkage evolution, pore structure, and mechanical performance under laboratory conditions. Several limitations should be acknowledged. The capillary pressure analysis is based on a simplified theoretical model with a constant contact angle assumption and was not directly validated experimentally. In addition, the carbonation treatment was conducted at laboratory scale, and its industrial applicability, energy consumption, and environmental impact were not evaluated. Moreover, long-term durability aspects, such as carbonation resistance and chloride penetration, remain to be further investigated. Therefore, additional studies integrating durability evaluation, and environmental assessment are still needed to better assess the engineering applicability of CRCP in cementitious materials.

## Figures and Tables

**Table 1:** Chemical composition of the used materials (wt.%).

	CaO	TiO_2_	SiO_2_	Al_2_O_3_	K_2_O	Fe_2_O_3_	MgO	Other
Cement	44.71	1.02	26.61	17.21	0.69	1.00	4.88	3.88
FA	3.52	1.81	57.00	26.10	1.30	4.20	0.68	5.39
CRCP	72.81	0.31	15.73	3.87	0.48	4.07	0.72	2.01

**Table 2:** Mix proportions of ternary mixtures (g).

Sample	Cement	Fly Ash	Sand	Water	CRCP
CR0	80	20	89	34	0
CR4	76.8	20	89	34	3.2
CR8	73.6	20	89	34	6.4
CR12	70.4	20	89	34	9.6
CR16	67.2	20	89	34	12.8

**Table 3:** Cumulative pore volume of each pore fraction (Z1–Z4) (mL/g).

Pore	CR0	CR4	CR8	CR12	CR16
Z1	2.26	2.87	2.98	2.65	2.36
Z2	1.41	1.09	1.16	1.18	1.25
Z3	1.60	1.35	1.14	1.57	1.59
Z4	1.20	1.11	1.05	1.05	1.11

**Table 4:** Pore structure characteristics of mortar as affected by CRCP content.

	Porosity	Total Pore Area	Med. Pore Diam. (Vol.)	Med. Pore Diam. (Area)	Avg. Pore Diam. (4 V/A)
	%	m^2^/g	nm	nm	nm
CR0	20.48	78.14	89.51	28.62	66.16
CR4	19.61	76.77	84.43	27.52	61.45
CR8	17.01	65.23	73.90	26.63	65.74
CR12	19.10	72.15	91.53	28.41	69.31
CR16	20.03	77.98	89.09	28.84	60.82

## Data Availability

The data presented in this study are available on request from the corresponding author.
